# Level and correlates of empathy and altruism during the Covid-19 pandemic. Evidence from a representative survey in Germany

**DOI:** 10.1371/journal.pone.0265544

**Published:** 2022-03-16

**Authors:** André Hajek, Hans-Helmut König

**Affiliations:** Department of Health Economics and Health Services Research, University Medical Center Hamburg-Eppendorf, Hamburg Center for Health Economics, Hamburg, Germany; Konkuk University, REPUBLIC OF KOREA

## Abstract

**Aim:**

Our purpose was to clarify the level and correlates of empathy and altruism in the German population during the Covid-19 pandemic.

**Methods:**

A nationally representative survey (n = 3,075) was conducted in August/September 2021. To measure empathy, a short scale based on the Interpersonality Reactivity Index (IRI; German version: Saarbrucken personality questionnaire, SPF) was used (SPF-K). Based on the International Personality Item Pool (IPIP; IPIP-5F30F-R1), the subscale altruism was used to quantify altruism.

**Results:**

The average altruism score was 3.3 (SD: 0.7), ranging from 1 to 5. Moreover, the average empathy score was 13.1 (SD: 2.8), ranging from 4 to 20. The level of empathy significantly differed between the subgroups. For example, high levels of empathy were identified among women (average: 13.7, SD: 2.7), individuals with children (average: 13.5, SD: 2.8), and individuals with migration background (average: 13.6, SD: 2.8). Effect sizes were mostly small. Similar differences (in terms of effect size) were identified between these groups regarding altruism. Additionally, regressions showed that higher levels of both empathy and altruism were associated with being female, younger age, having children, sports activities and having at least one chronic disease. Moreover, vaccination against Covid-19 was only associated with higher altruism, but not with higher empathy.

**Conclusion:**

Our study emphasized the moderately high level of empathy and altruism in Germany during times of the pandemic. Identifying the correlates of these factors may help to address individuals with very low levels of these factors.

## Introduction

Altruism generally refers to disinterestedness and selflessness [1]. Individuals with a low level of altruism do not actively care about the well-being of others, while people with a high level of altruism put the needs of others above their own and help others. Empathy refers to the ability to imagine what life is like for another individual [2].

Previous research has demonstrated that both factors (i.e., altruism and empathy) are associated with positive outcomes [3] (e.g., higher relationship quality [4], less prejudice [5] or greater social competence [6]). Additionally, a systematic review has shown that altruism is a main driver of donating blood [7]–similarly, empathy is also an important motivator for donating blood [8]. As recently shown in empirical studies, we assume that such “value-based”(i.e., empathy and altruism) [8] motives for donating blood may also be applied to wearing face masks during the current pandemic [9] and vaccination against Covid-19 [10–12]. While individuals can protect themselves via the vaccination against Covid-19 (for an overview regarding the efficacy of different approved vaccines, please see [13]), they can also protect others by reducing the likelihood of transmission (community protection [10]). Moreover, wearing face masks is effective in reducing the transmission of Covid-19 [14]. Thus, we assume that altruism and empathy are important factors in the fight against the Covid-19 pandemic (including factors such as keeping the distance and avoiding social contacts). For this reason, it is of importance to identify the level and correlates of empathy and altruism in the German population during the Covid-19 pandemic. Identifying the correlates of both factors may assist in addressing individuals with very low levels of these factors.

## Materials and methods

### Sample

Data were taken from an online survey (nationally-representative, n = 3,075 respondents, from late August to early September 2021). Individuals from 18 years to 70 years living in Germany were included. A well-established market research company recruited the individuals based on an online panel. The recruitment was done in a way that it reflects the distribution of age range, gender and federal state in the adult population in Germany (i.e., quota sampling; quotas were used from the best4planning 2020). Based on the socio-demographic data, a random sample from the population of the online access panel was drawn. For our current study, about 14,000 participants were contacted in order to take part in this study.

Participants provided their informed consent. Our study was approved by the Local Psychological Ethics Committee of the Center for Psychosocial Medicine (LPEK) of the University Medical Center Hamburg-Eppendorf (number: LPEK-0356).

### Outcomes

Altruism was quantified using the subscale ‘altruism’ of the International Personality Item Pool (IPIP; more precisely: IPIP-5F30F-R1 [15]: International Personality Item Pool with five factors and 30 facets, revised version 1). The German version was validated recently [16]. This subscale consists of six items (in each case: 1 = strongly agree, 2 = agree, 3 = neutral, 4 = disagree, 5 = strongly disagree). For example: “I have a good word for everyone”. All items were recoded. Thereafter, all recoded items were averaged to build the final score which ranges from 1 to 5 (higher scores indicate higher altruism). In our study, Cronbach’s alpha was .87.

Empathy was quantified using a short scale based on the Interpersonality Reactivity Index (IRI [2]; German version: Saarbrucken personality questionnaire, SPF [17] called SPF-K (K for the German word “Kurz”–referring to the shortness) [18]). It consists of four items (in each case: 1 = never, 2 = rarely, 3 = sometimes, 4 = often, 5 = always) (please see Paulus for further details [18]. In accordance with the suggestion given by Paulus [18], a sum score was computed (ranging from 4 to 20, with higher values reflecting higher empathy). In our study, Cronbach’s alpha was .81.

### Independent variables

Correlates were included as follows in regression analysis: sex, age, marital status (married, living together with spouse; married, not living together with spouse; single; widowed; divorced), level of education (upper secondary school; qualification for applied upper secondary school; polytechnic Secondary School; intermediate Secondary School; Lower Secondary School; currently in school training/education; without school-leaving qualification), and occupational status (full-time employed; retired; other), children in own household (no; yes), migration background (no; yes), and employment status (full-time employed; retired; other). Furthermore, these lifestyle-related correlates were included in regression analysis: sports activities (no sports activity; less than one hour a week; regularly, 1–2 hours a week; regularly, 2–4 hours a week; regularly, more than 4 hours a week), smoking status (yes, daily; yes, sometimes; no, not anymore; never smoker), and alcohol intake (daily; several times per week; once a week; 1–3 times per month; less often; never). Furthermore, these health-related correlates were included: vaccination against Covid-19 (no; yes), presence of one or more chronic conditions (no; yes) and self-rated health (single item: from 1 = very bad to 5 = very good).

### Statistical analysis

Levels of empathy and altruism for several selected subgroups were first displayed stratified by sex, age groups, educational level, having children, migration background, vaccination against Covid-19, and chronic diseases. Subsequently, multiple linear regressions were used to identify the sociodemographic, lifestyle-related and health-related covariates of empathy and altruism.

Statistical significance was defined as p value of 0.05 or smaller. Stata 16.1 (Stata Corp., College Station, Texas) was used to perform statistical analyses.

## Results

### Sample characteristics

In our sample, 51.1% of the participants were female and mean age equaled 44.5 years (standard deviation, SD: 14.8 years; 18–70 years). The average altruism score was 3.3 (SD: 0.7), ranging from 1 to 5 (see Table 1). Moreover, the average empathy score was 13.1 (SD: 2.8), ranging from 4 to 20. The level of empathy significantly differed between the subgroups. For example, high levels of empathy were identified among women (average: 13.7, SD: 2.7; Cohen’s d was .39 for the difference between women and men), individuals with children (average: 13.5, SD: 2.8; Cohen’s d was .17 for the difference between individuals with children and individuals without children), and individuals with migration background (average: 13.6, SD: 2.8; Cohen’s d was .18 for the difference between individuals with migration background and individuals without migration background). Similar differences (in terms of effect size) were identified between these groups regarding altruism. Further details are given in Table 1.

**Table 1:** Level of empathy and altruism among several groups.

	n	Level of empathy	p-value	Level of altruism	p-value
Total sample	3,075	13.1 (2.8)		3.3 (0.7)	
Gender			< .001		< .001
Male	1,502	12.6 (2.8)		3.2 (0.7)	
Female	1,570	13.7 (2.7)		3.4 (0.7)	
Diverse	3	13.3 (2.5)		3.0 (0.6)	
Age group			< .001		< .001
18 to 29 years	628	13.6 (2.9)		3.4 (0.7)	
30 to 39 years	597	13.0 (2.8)		3.3 (0.7)	
40 to 49 years	597	13.2 (2.9)		3.3 (0.7)	
50 to 59 years	659	13.0 (2.8)		3.3 (0.6)	
60 years and older	594	12.8 (2.7)		3.1 (0.7)	
Children in own household			< .001		< .001
No	2,206	13.0 (2.8)		3.2 (0.7)	
Yes	869	13.5 (2.8)		3.4 (0.7)	
Marital status			.12		< .01
Single/Divorced/Widowed/Married, not living together with spouse	1,313	13.0 (2.8)		3.3 (0.7)	
Married, living together with spouse	1,762	13.2 (2.8)		3.3 (0.7)	
Education			< .001		.40
upper secondary school	1326	13.3 (2.7)		3.3 (0.6)	
qualification for applied upper secondary school	328	13.1 (2.7)		3.3 (0.6)	
polytechnic Secondary School	168	12.8 (2.9)		3.2 (0.7)	
intermediate Secondary School	888	13.1 (2.9)		3.3 (0.7)	
Lower Secondary School	347	12.6 (3.1)		3.2 (0.7)	
currently in school training/education	9	15.8 (3.3)		3.4 (0.8)	
without school-leaving qualification	9	13.8 (2.2)		3.1 (0.5)	
Migration background			< .01		.85
No	2,724	13.1 (2.8)		3.3 (0.7)	
Yes	351	13.6 (2.8)		3.3 (0.7)	
Employment status			< .001		< .001
Full-time employed	1,458	12.9 (2.8)		3.3 (0.7)	
Retired	499	12.8 (2.9)		3.2 (0.7)	
Other	1,118	13.6 (2.8)		3.4 (0.7)	
Chronic diseases			.22		.43
Absence of at least one chronic disease	1,765	13.1 (2.9)		3.3 (0.7)	
Presence of at least one chronic disease	1,310	13.2 (2.8)		3.3 (0.7)	
Vaccinated against Covid-19			.98		< .01
No	593	13.1 (3.1)		3.2 (0.7)	
Yes	2,482	13.1 (2.8)		3.3 (0.7)	

Notes: Independent t-tests or oneway anovas were conducted, as appropriate.

### Regression analysis

Results of multiple linear regressions are given in Table 2 (unstandardized beta-coefficients (β) and p-values (p) are given in this section). Higher levels of empathy were associated with being female (β = .93, p < .001), younger age (30 to 39 years compared to 18 to 29 years, β = -.38, p < .05), having children (β = .38, p < .01), educational level (in both directions), having a migration background (β = .35, p < .05), other employment status (compared to full-time employed, β = .36, p < .01), smoking daily (compared to never smokers, β = .32, p < .05), sports activities (e.g., regularly, 2–4 hours a week compared to no sports activity, β = .96, p < .001) and having at least one chronic disease (β = .40, p < .001).

**Table 2:** Correlates of empathy and altruism. Results of multiple linear regressions.

Independent variables	Empathy	Altruism
Sex:—Women (Ref.: Men)	0.93***	0.18***
	(0.11)	(0.03)
- Diverse	0.49	-0.28
	(1.03)	(0.27)
Age group: 30 to 39 years (Ref.: 18 to 29 years)	-0.38*	-0.12**
	(0.17)	(0.04)
- 40 to 49 years	-0.07	-0.14**
	(0.18)	(0.04)
- 50 to 59 years	-0.20	-0.20***
	(0.18)	(0.04)
- 60 years and older	-0.04	-0.29***
	(0.21)	(0.05)
Marital status:—Married, living together with spouse (Ref.: Single/Divorced/Widowed/Married, not living together with spouse)	0.15	0.07*
	(0.11)	(0.03)
Children in own household:—Yes (Reference: No)	0.38**	0.14***
	(0.12)	(0.03)
Highest educational degree:—qualification for applied upper secondary school (Ref.: upper secondary school)	-0.19	0.03
	(0.16)	(0.04)
- polytechnic Secondary School	-0.21	0.08
	(0.24)	(0.06)
- intermediate Secondary School	-0.11	0.07*
	(0.13)	(0.03)
- lower Secondary School	-0.38*	0.07
	(0.19)	(0.05)
- currently in school training/education	2.53*	0.08
	(1.07)	(0.25)
- without school-leaving qualification	0.41	-0.20
	(0.73)	(0.17)
Migration:—Migration background (Ref.: no migration background)	0.35*	-0.06
	(0.16)	(0.04)
Employment status:—Retired (Ref.: Full-time employed)	0.06	0.02
	(0.19)	(0.04)
- Other	0.36**	0.02
	(0.12)	(0.03)
Smoking:—Yes, daily (Ref: Never smoker)	0.32*	0.04
	(0.14)	(0.03)
- Yes, sometimes	0.27	-0.02
	(0.19)	(0.05)
No, not anymore	-0.07	-0.05
	(0.13)	(0.03)
Sports activities:—Less than one hour a week (Ref.: no sports activity)	0.20	0.08*
	(0.15)	(0.03)
- Regularly, 1–2 hours a week	0.63***	0.10**
	(0.15)	(0.03)
- Regularly, 2–4 hours a week	0.96***	0.13**
	(0.16)	(0.04)
- Regularly, more than 4 hours a week	0.83***	0.12**
	(0.18)	(0.04)
Alcohol intake:—Daily (Ref.: Never)	-0.01	-0.05
	(0.26)	(0.06)
- Several times a week	-0.26	0.00
	(0.17)	(0.04)
- Once a week	-0.02	-0.01
	(0.17)	(0.04)
- 1–3 times a month	-0.18	-0.00
	(0.17)	(0.04)
- Less often	-0.11	-0.00
	(0.16)	(0.04)
Chronic diseases: Presence of at least one chronic disease (Absence of chronic diseases)	0.40***	0.08**
	(0.11)	(0.03)
Self-rated health (1 = very bad to 5 = very good)	0.07	-0.02
	(0.07)	(0.02)
Vaccinated against Covid-19: Yes (Ref.: No)	0.02	0.11***
	(0.14)	(0.03)
Constant	11.65***	3.11***
	(0.38)	(0.09)
Observations	3,075	3,075
R^2^	0.07	0.07

Beta-coefficients (unstandardized) are displayed; robust standard errors in parentheses;

*** p<0.001,

** p<0.01,

* p<0.05,

^+^ p<0.10.

**Fig 1:**
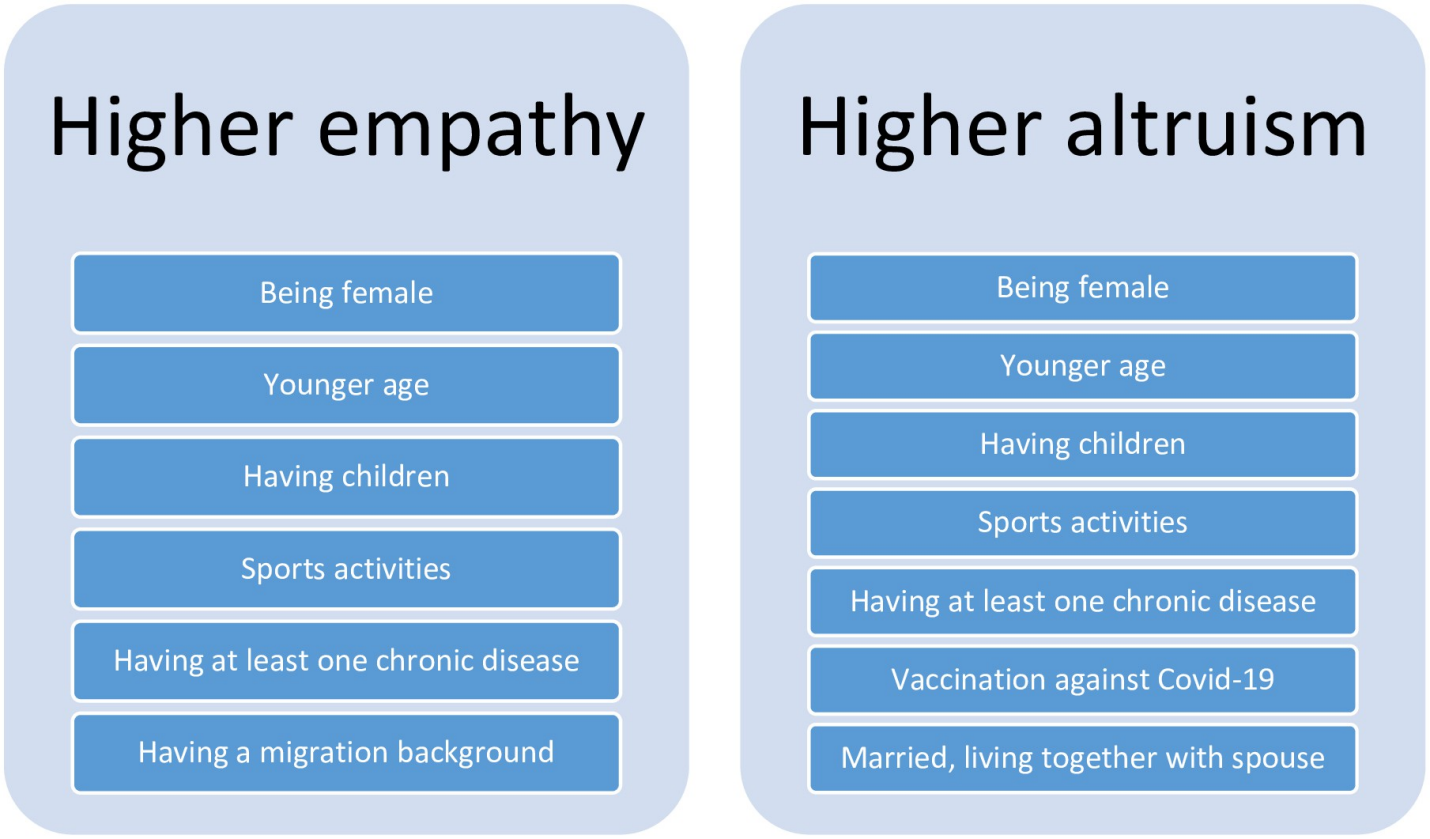
Some key findings (based on regression analyses).

Furthermore, regressions showed that higher levels of altruism were associated with being female (β = .18, p < .001), younger age (e.g., 60 years and older compared to 18 to 29 years, β = -.29, p < .001), being married (β = .07, p < .05), having children (β = .14, p < .001), lower education (intermediate Secondary School compared to upper Secondary School, β = .07, p < .05), sports activities (e.g., regularly, 2–4 hours a week compared to no sports activity, β = .13, p < .01), being vaccinated against Covid-19 (β = .11, p < .001), and having at least one chronic disease (β = .08, p < .01). The key findings of regression analyses are also displayed in Fig 1.

## Discussion

Our goal was to clarify the level and correlates of empathy and altruism in the German adult population in times of the Covid-19 pandemic. The level of empathy significantly differed between the subgroups (effect sizes were mostly small). For example, high levels of empathy were identified among women, individuals with children, and individuals with migration background. Similar differences (in terms of effect size) were identified between these groups regarding altruism. Regressions showed that higher levels of both empathy and altruism were associated with being female, younger age, having children, sports activities and having at least one chronic disease. Further correlates were identified. This is the first study clarifying the level and correlates of altruism and empathy in the general adult population during the Covid-19 pandemic and thus extends our current knowledge.

Quite high levels of empathy and altruism have also been reported in other countries and specific groups during the pandemic (e.g., [19, 20]). However, it should be noted that reference values are missing.

In contrast to a recent meta-analysis (conducted prior to the pandemic) which identified a positive association between age and altruism and in contrast to a recent study (performed prior to the pandemic) showing increases in empathy across the life span (particularly after age 40) in different cohorts [21], we identified higher altruism and empathy levels among younger adults [22]. Our findings may be explained by the fact that particularly younger adults reported high depression and anxiety levels in Germany during the pandemic [23]. Thus, younger adults may particularly suffer mentally because of the pandemic and may think that prosocial behavior such as empathy and altruism (e.g., avoiding social contacts) are required to overcome this pandemic together. However, future research is required to test our assumptions.

The differences between women and men regarding empathy and altruism found in our study are quite plausible. Such differences are often attributed to actual sex differences in empathy and self-confidence (i.e., women make more altruistic and empathic choices rather than egoistic choices), but also by confirmation to gender ideals (i.e., women are seen as empathic and caring) or self-identification processes (i.e., they may wish to appear empathic and altruistic according to their own internal audience) [24].

It has repeatedly been shown that particularly individuals with children reported adverse outcomes during the pandemic–for example due to school closings (e.g., parental well-being or perceived stress [25, 26]). Hence, they may know how to put their own needs aside and take care of the children and work at the same time during the pandemic. Therefore, individuals with children may eagerly and impatiently wait for the pandemic to finally be over and may thus behave very empathically and altruistically (e.g., avoid social contacts whenever they can) to accelerate this process. Furthermore, and more generally, since they have children, they may know how to put the needs of children above their own and help them.

In accordance with our previous thoughts, individuals with at least one chronic disease may be particularly vulnerable to a severe course of COVID-19. Thus, they may also behave altruistically and empathically (i.e., reducing social contacts or keeping the distance) to speed up the process of overcoming the pandemic. Moreover, since those individuals are sometimes particularly dependent on the support of others, they themselves may develop empathy and altruism. However, future research in this area is required.

In our study, the association between more sports activities and both higher altruism and higher empathy may, for example, be explained by performing team sports activities which has been shown to be associated with higher empathy [27]. However, it has also been shown that individual sport is associated with higher empathy (compared to no sports activities) [27]. Thus, it is assumed that the amount of sports can assist in increasing our outcomes [27].

Individuals with a migration background also reported higher levels of empathy. One way to explain such an association may be that individuals with a migration background already have managed to overcome obstacles (e.g., refugee experience). In turn, such experiences may strengthen the feeling of empathy–particularly in difficult times. However, future research in this area is urgently required.

Interestingly and quite unexpectedly, vaccination against Covid-19 was only associated with higher altruism, but not with higher empathy in our study. This is in contrast to a previous study showing an association between higher empathy and intentions to vaccinate against Covid-19 [11] in the United Kingdom in September 2020 (thus, prior to the availability of a Covid-19 vaccine). Differences in the variables (intentions to vaccine vs. actual vaccination in our study) may explain these discrepancies. Moreover, vaccination prioritization took place in Germany until summer 2021. The association between vaccination against Covid-19 and higher altruism may be simply explained by the fact that the vaccination can also protect others by reducing the likelihood of transmission.

Some strengths and limitations are worth acknowledging. Data were taken from a sample covering the general adult population in Germany. Reliable tools were used to quantify both altruism and empathy in times of the pandemic. Nevertheless, it should be acknowledged that these are quite short tools. Consequently, upcoming research (e.g., using the SPF [17]) is required to confirm our findings. Moreover, longitudinal studies are required to clarify the directionality between the correlates and empathy as well as altruism.

In conclusion, our study emphasized the moderately high level of empathy and altruism in Germany during times of the Covid-19 pandemic. Identifying the correlates of these factors (e.g., being male, higher age) may help to address individuals with very low levels of these factors.
